# Differential Responses of Digesta- and Mucosa-Associated Jejunal Microbiota of Hu Sheep to Pelleted and Non-Pelleted High-Grain Diets

**DOI:** 10.3390/ani12131695

**Published:** 2022-06-30

**Authors:** Zhiqiang Zhong, Yuning Zhang, Xiaotong Li, Lingyun Li, Ruiyang Zhang, Shuyi Zhang

**Affiliations:** College of Animal Science and Veterinary Medicine, Shenyang Agricultural University, Shenyang 110866, China; zhongzhiqiang@stu.syau.edu.cn (Z.Z.); zhangyuning@stu.syau.edu.cn (Y.Z.); lixiaotong20221019@163.com (X.L.); lilingyun@stu.syau.edu.cn (L.L.)

**Keywords:** jejunum, digesta, mucosa, microbial composition, Hu sheep

## Abstract

**Simple Summary:**

Currently, feeding ruminants high-grain (HG) diets is a common feeding pattern, but this practice can have detrimental effects on these animals in the form of altering their gut microbiota. The aim of this study was to explore the structural and functional responses of jejunal digesta- and mucosa-associated microbiota to the low-grain as well as non-pelleted and pelleted high-grain (HP) diets. The results indicated that different diets altered the composition of digesta- and mucosa-associated microbiota, and the abundance of acid-producing bacteria was increased in both jejunal digesta and mucosa after the HG and HP diets. Meanwhile, the HP diets somewhat changed the impact of the HG diets, as HP diets reduced the proliferation of pro-inflammatory bacteria in the jejunal digesta and mucosa when compared to HG diets.

**Abstract:**

In the present study, we utilized 16S rRNA sequencing to uncover the impacts of non-pelleted (HG) or high-grain pelleted (HP) diets on the microbial structure and potential functions of digesta- and mucosa-associated microbiota in the jejunum of Hu sheep. Here, we randomly assigned 15 healthy male Hu sheep into three groups and fed the control diets (CON), HG, and HP diets, respectively. The experiment period was 60 days. The HP diets had the same nutritional ingredients as the HG diets but in pelleted form. At the finish of the experiment, the jejunal digesta and mucosa were gathered for microbial sequencing. The results of PCoA and PERMANOVA showed that different dietary treatments had significant impact (*p* < 0.05) on digesta- and mucosa-associated microbiota in the jejunum of Hu sheep. For specific differences, HG diets significantly increased (*p* < 0.05) the abundance of some acid-producing bacteria in both jejunal digesta (*Bifidobacterium*, OTU151, and OTU16) and mucosa (*Rikenellaceae RC9 gut group*, and *Bifidobacterium*) of Hu sheep compared with the CON diets. Besides the similar effects of the HG diets (increased the acid-producing bacteria such as *Olsenella*, *Pseudoramibacter*, and *Shuttleworthia*), our results also showed that the HP diets significantly decreased (*p* < 0.05) the abundance of some pro-inflammatory bacteria in the jejunal digesta (*Mogibacterium*, and *Marvinbryantia*) and mucosa (*Chitinophaga*, and *Candidatus Saccharimonas*) of Hu sheep compared with the HG diets. Collectively, these findings contributed to enriching the knowledge about the effects of HG diets on the structure and function of intestinal microbiota in ruminants.

## 1. Introduction

In the modern feed industry, the high-grain (HG) diets have been commonly used to sustain the growth performance of fattening ruminants [1,2]. However, the long-termed use of HG diets has been reported to generate a flood of adverse effects on the host [3], such as reshaping microbial composition, as indicated by the reduced microbial diversity and increased starch degrader and pro-inflammatory bacteria in gastrointestinal tract (GI). Meanwhile, HG diets also caused the accumulation of pro-inflammatory metabolites, and injured mucosal cells in the GI [4,5,6,7,8]. Therefore, the systemic effects of HG diets on a ruminant host have received continuous attention in recent years.

With the widespread use of HG diets, the pelleted HG diets have gradually attracted much attention. Previous studies have shown that pelleted diets improved the utilization of nutrition [9,10], prevented selective eating behavior, and ensured a balanced nutritional intake, thus improving farming benefits [11,12,13]. Trabi et al. (2019) demonstrated that, in addition to having similar effects as HG diets, high-grain pelleted (HP) diets also resulted a greater abundance of simple sugar fermenters (such as *Megasphaera*) in the rumen of Hu sheep [14]. Lin et al. (2021) reported that compared to HG diets, HP diets specifically increased the abundance of some acetate-producing bacteria (such as *Ruminococcus*) and reduced the abundance of some starch-degrader bacteria (such as *Roseburia* spp.) in the colon of Hu sheep [15]. However, current studies have focused on the rumen and hindgut, there were little information about the effects of HP diets on the small intestine of ruminants.

In addition to the rumen and the hindgut, the micro-ecological environment in the small intestine plays a crucial role in the digestion of nutrients and organism health for ruminants [16,17]. For instance, the microbial variation in the jejunum has been demonstrated to be a factor influencing the feed efficiency of steers [16]. Meanwhile, diets affected the intestinal micro-ecological balance. Previous studies demonstrated that HG diets resulted in histological injures and altered the microbial structure in jejunum of goats [18]. Significant differences were seen in the colonization and distribution of intestinal microbiota in the ecological niches (digesta or mucosa), and the mucosa-associated microbiota was found to be of greater importance in host immunity and health compared to the digesta-associated microbiota [19,20]. Hence, studying the microbial community in different ecological niches could provide us with a more comprehensive understanding of changes in the intestine in response to different diets. However, to our knowledge, there are no reports that simultaneously revealed the effects of HP diets on the jejunal digesta- and mucosa-associated microbiota in ruminants.

This study used 16S rRNA sequencing to compare the impact of low-grain, HG, and HP diets on changes in digesta- and mucosa-associated microbiota and their underlying metabolic functions of in the jejunum of Hu sheep.

## 2. Materials and Methods

### 2.1. Experimental Design and Animals

All animal experiments complied with the guidelines of the Experimental Animal Welfare Ethics Committee of Shenyang Agricultural University. This research was a portion of systematic studies exploring the impacts of high-grain diets on the gastrointestinal tract fitness of Hu sheep, and experimental design, animal rearing and diets have been published elsewhere [14,21]. In brief, a total of 15 male and healthy Hu sheep with similar body weight (26.80 ± 0.32 kg) and age (about 180 days) were assigned into the following three dietary groups: the control diets (CON: 30% concentrate + 70% forage), the non-pelleted high-grain diets (HG: 70% concentrate + 30% forage), and the pelleted high-grain diets (HP: 70% concentrate + 30% forage). The concentrate in the CON and HG groups were in mashed form, and the pelleted high-grain diets (HP) had the same ingredients as the HG diets but in pelleted form. A pelleting machine (Jiangsu Muyang Group Co., Ltd., Yangzhou, China) was used to pellet the feed (3.2 mm diameter) at 70 °C. At the preliminary feeding period, all animals were fed the same diet containing alfalfa hay and oat straw for 18 days. The total experiment period was 60 days and all experimental animals were reared in individual pens and fed diets ad libitum. The detailed ingredients and nutrient composition of treatment diets were listed in Table S1.

### 2.2. Sample Collection

On day 60, all sheep were euthanized and butchered 4–5 h after the morning feeding. After slaughter, the abdominal cavity of experimental animals was quickly dissected, and the jejunum and ileum were ligated with cotton thread and separated. The contents and mucosa (scraped from the ileal tissue using sterile slides) of the jejunum were homogenized and blended completely, and then typical samples were gathered. Samples were packed in sterile centrifuge tubes, quickly frozen in liquid nitrogen, and put away in a −80 °C refrigerator until isolation of microbial DNA.

### 2.3. Genomic DNA Isolation

Samples were fully thawed, homogenized, and 0.3 g samples were used for microbial DNA isolation. According to the standard procedure of the manual, we used the QIAamp DNA stool Mini Kit (QIAGEN, Hilden, Germany) to isolate the genomic DNA in the jejunal mucosa and contents samples. After isolation, DNA samples were further assessed by a NanoDrop spectrophotometer (Nyxor Biotech, Paris, France). Only high-quality DNA samples were used for subsequent PCR amplification and sequencing.

### 2.4. Illumina Sequencing and Data Analysis

The following bacterial universal primers 338F (5′-ACTCCTRCGGGAGGCAGCAG-3′) and 806R (5′-GGACTACCVGGGTATCTAAT-3′) were adopted to conduct the amplification of 16S rRNA V3-V4 region of jejunal mucosa and contents samples [22]. PCR amplification was conducted following the standard procedures, with three replicates for each sample and a final volume of 20 μL mixture. Subsequently, the PCR products of one sample were mixed thoroughly and detected using 2% agarose gels. Then, the purification was conducted using AxyPrep DNA Gel Extraction Kit (Axygen Biosciences, Union City, CA, USA). Illumina sequencing library was created and then sequenced on the Illumina MiSeq platform.

The effective sequences of all samples were obtained according to the barcode, and then performed quality control and filtering on sequenced reads. According to the overlapping relationship between reads, the sequenced paired reads were combined using FLASH (version 1.2.7), and the lowest overlap length was 10 bp [23]. The Chimera removal process was conducted using the UCHIME algorithm in the USEARCH package [24]. We conducted the taxonomic analysis of representative OTU sequences at the 97% similar level by the Ribosomal Database Project (RDP) classifier (https://sourceforge.net/projects/rdp-classifier/ (accessed on 12 November 2021)), and the classification information of each OTU was obtained. The SILVA database (http://www.arb-silva.de (accessed on 12 November 2021)) was adopted to conduct the alignment of 16S rRNA sequences. The rarefaction curves were calculated to evaluate the sampling effort. Estimators of Chao 1, Shannon index, and Simpson were generated through QIIME (http://1iime.org/ (accessed on 15 November 2021)) and were used for evaluating the microbial community richness and diversity.

### 2.5. The Prediction of Microbial Functional Genes

Phylogenetic investigation of communities by reconstruction of unobserved states (PICRUSt) is commonly used and reliable biological information software that uses as 16S rRNA genes for predicting the metabolic functions of microorganisms [25]. In the present study, for metagenome prediction, OTUs that contain identifiers that match tips from the marker gene from Greengenes 16S rRNA gene database were picked using QIME 1.8.0 [26]. To obtain high predictive results, the resulting OTU table was normalized by 16S copy number and then utilized for metagenome inference of KEGG ortholog via the software PICRUSt. Differences in the microbial functions of the jejunum mucosa and contents among different dietary treatments were measured by the combined analysis of PCoA and PERMANOVA.

### 2.6. Statistical Analysis

All experimental data from this experiment were statistically analyzed using SPSS (SPSS v. 20.0, SPSS Inc., Chicago, IL, USA). The Bray–Curtis distance based principal coordinate analysis (PCoA) was performed to determine dissimilarities in microbial community, and the significant differences between different groups were assessed through the permutation multivariate analysis (PERMANOVA). Featured OTUs and metabolic pathways were determined for each treatment group through a linear discriminant analysis effect size (LEfSe) analysis. The discrepant significance at genus among these 3 groups was shown by the heat map.

## 3. Results

### 3.1. Overview of Jejunal Digesta- and Mucosa-Associated Microbiota

Using 16S rRNA sequencing, 693,424 valid reads (46,228 valid reads for each sample on average) from digesta samples, and 698,373 valid reads (46,558 valid reads for each sample on average) from mucosal samples were obtained for this study (Figure S1). The results of rarefaction curves generated from digesta- and mucosa-associated microbiota indicated that sequencing reads approached a plateau at 30,825 and 33,924 reads, respectively.

**Figure 1:**
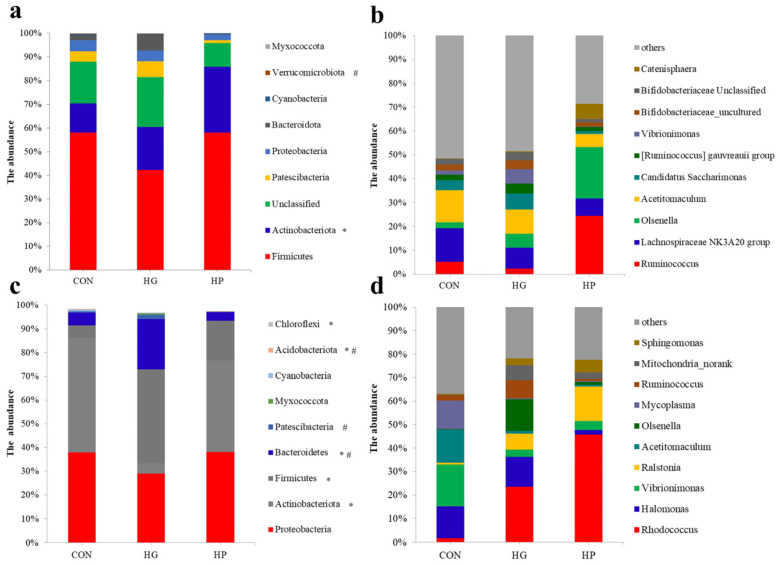
Taxonomic analysis of jejunal digesta- and mucosa-associated microbiota in Hu sheep (n = 5) fed CON, HG and HP diets at different taxonomic levels. The composition of the digesta-associated microbiota at the phylum (**a**) and genus (**b**) levels. The composition of the mucosa-associated microbiota at the phylum (**c**) and genus (**d**) levels. * indicates that there has a significant difference between the CON and HG groups; # indicates that there has a significant difference between the HG and HP groups. CON, the control group; HG, the high-grain group; HP, the pelleted high-grain group.

For the jejunal digesta samples, regardless of the dietary treatments, the most predominant phyla (Figure 1a) were *Firmicutes* (average 52.84%), *Actinobacteriota* (average 19.36%), and *Patescibacteria* (average 4.06%). At the genus level (Figure 1b), the five most predominant genera were *Ruminococcus* (average 10.63%), *Lachnospiraceae NK3A20* group (average 10.10%), *Olsenella* (average 9.92%), *Acetitomaculum* (average 9.69%), and *Candidatus Saccharimonas* (average 4.05%). For the jejunal mucosal samples, the most predominant phyla (Figure 1c) in the three treatment groups were *Proteobacteria* (average 35.06%), *Actinobacteriota* (average 30.37%), and *Firmicutes* (average 20.48%). At the genus level (Figure 1d), regardless of the dietary treatments, *Rhodococcus* (average 23.61%), *Halomonas* (average 9.40%), *Vibrionimonas* (average 8.25%), *Ralstonia* (average 7.38%), and *Acetitomaculum* (average 5.38%) were the five most predominant genera in the mucosa-associated microbiota of Hu sheep.

### 3.2. Diversity and Richness of Jejunal Microbiota

**Figure 2:**
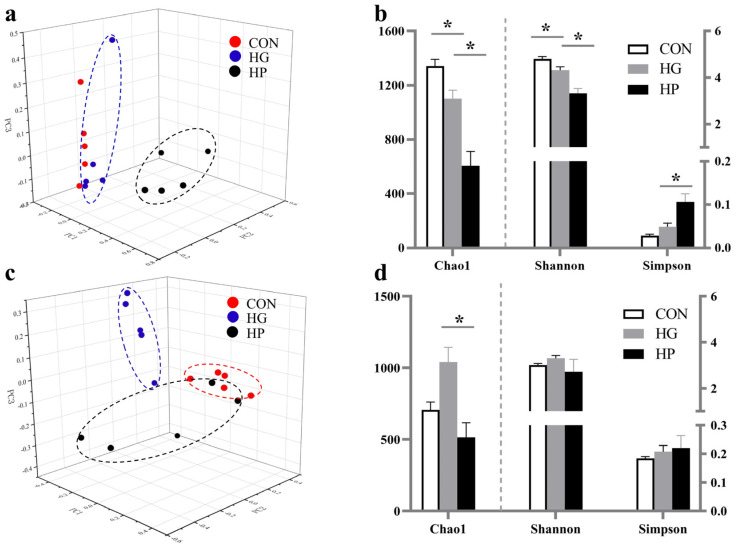
The alterations in the microbial diversity and richness of jejunal digesta and mucosa in Hu sheep (n = 5) fed the CON, HG and HP diets. The principal coordinate analysis (PCoA) of the jejunal digesta- (**a**) and mucosa- (**c**) associated microbiota. Summary overview of the Chao1, Shannon, and Simpson index in the jejunal digesta- (**b**) and mucosa- (**d**) associated microbiota. CON, the control group; HG, the high-grain group; HP, the pelleted high-grain group. * *p* < 0.05.

Regarding the digesta-associated microbiota, the PCoA and PERMANOVA (Figure 2a) indicated the HG diets had no significant difference compared to the CON diet (PERMANOVA *p* = 0.105), but a significant difference was observed between the HP and HG diets (PERMANOVA *p* = 0.008). An analysis of α-diversity showed that the Chao 1 and Shannon index values of the HG diets were lower (*p* < 0.05; Figure 2b) than in the CON diet, and there was a significant decrease in Chao 1 and Shannon index value (*p* < 0.05) in the HP diets compared with the HG diet. In addition, the HP diet had a higher Simpson index value (*p* < 0.05) compared to the HG diets was also found in the present study.

Regarding the mucosa-associated microbiota, a PCoA and PERMANOVA revealed significant differences between the CON and HG diets (PERMANOVA *p* = 0.01; Figure 2c), and between the HG and HP diets (PERMANOVA *p* = 0.026). Moreover, the results of the microbial richness and diversity estimators suggested that the HP diet significantly reduced the Chao 1 index value compared with the HG diet (*p* = 0.027; Figure 2d), while there were no significant differences in the Shannon and Simpson index value among the three groups.

### 3.3. Alterations in the Microbial Composition of the Jejunal Digesta

**Figure 3:**
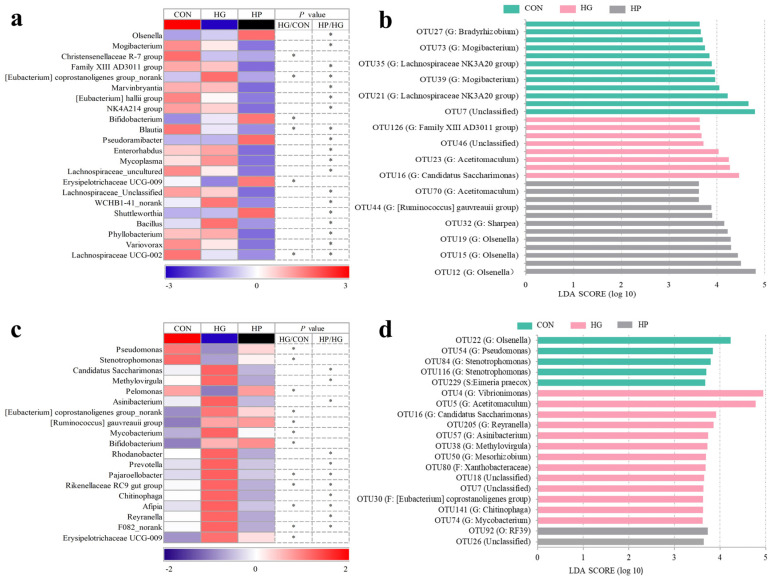
Statistical analysis of jejunal digesta- and mucosa-associated microbiota at the genus (**a**,**c**) and OTU (**b**,**d**) levels in Hu sheep (n = 5) fed the CON, HG, and HP diets. CON, the control group; HG, the high-grain group; HP, the pelleted high-grain group. * *p* < 0.05.

Next, the microbial changes in the jejunal digesta and mucosa were investigated at different taxonomic levels. For the digesta-associated microbiota, at the phylum level, there was a lower abundance (*p* < 0.05) of Firmicutes in the HG group compared to the CON group, and a significantly lower abundance (*p* < 0.05) of Verrucomicrobiota in the HP group compared to the HG group. At the genus level (Figure 3a), there was an increased abundance of *Bifidobacterium* and *Eubacterium coprostanoligenes* group_norank and a reduced abundance (*p* < 0.05) of *Christensenellaceae R-7 group*, *Blautia*, *Erysipelotrichaceae UCG-009*, and *Lachnospiraceae UCG-002* in the HG group than the CON group. Moreover, we also observed higher abundance (*p* < 0.05) of *Olsenella, Pseudoramibacter*, and *Shuttleworthia* and a reduced abundance (*p* < 0.05) of *Mogibacterium*, *Family XIII AD3011 group*, *Marvinbryantia*, *Eubacterium hallii group*, *NK4A214 group*, *Blautia*, and *Bacillus* in the HP group compared to the HG group.

At the OTU level (Figure 3b), we determined the characteristic microbiota in the treatment groups using LEfSe analysis. We found that 32 OTUs were influenced after the dietary treatment (*p* < 0.05). Among them, 12 OTUs, including OTU25 (Unclassified), OTU35 (G: *Lachnospiraceae NK3A20 group*), OTU40 (G: *Lachnospiraceae NK3A20 group*), OTU55 (G: *Lachnospiraceae NK3A20 group*), OTU27 (G: *Bradyrhizobium*), OTU56 (G: *Saccharofermentans*), and OTU73 (G: *Mogibacterium*) were enriched (*p* < 0.05) in the CON group. In the HG group, OTU16 (G: *Candidatus Saccharimonas*) and OTU126 (G: *Family XIII AD3011 group*) were the featured microorganisms of jejunal contents (*p* < 0.05). Moreover, OTU32 (G: *Sharpea*), OTU42 (S: *Bifidobacterium choerinum*), OTU44 (G: *Ruminococcus gauvreauii group*), and OTU59 (G: *Catenisphaera*) were enriched (*p* < 0.05) in the HP group.

### 3.4. Alterations in the Microbial Composition of the Jejunal Mucosa

Regarding the mucosa-associated microbiota, at the phylum level, there was a decreased abundance (*p* < 0.05) of Actinobacteriota, Chloroflexi, and Acidobacteriota, while an increased abundance (*p* < 0.05) of Firmicutes and Bacteroidetes in the HG group compared to the CON group. The abundance of Acidobacteriota increased significantly (*p* < 0.05), and the abundance of Bacteroidetes and Patescibacteria decreased significantly (*p* < 0.05) in the HP group compared to the HG group. In terms of genus-specific differences (Figure 3c), a higher abundance (*p* < 0.05) of the *Eubacterium coprostanoligenes* group_norank, *Ruminococcus gauvreauii group*, *Mycobacterium*, *Bifidobacterium*, *Pajaroellobacter*, *Rikenellaceae RC9 gut group*, and *Erysipelotrichaceae UCG-009*, and a lower abundance (*p* < 0.05) of *Pseudomonas*, *Stenotrophomonas*, and *Pelomonas* were found in the HG group compared to the CON group. Moreover, a manifest reduction (*p* < 0.05) in the abundance of *Candidatus Saccharimonas*, *Methylovirgula*, *Asinibacterium*, *Rhodanobacter*, *Prevotella*, *Pajaroellobacter*, *Rikenellaceae RC9 gut group*, *Chitinophaga*, and *Reyranella* was found in the HP group compared with the HG group.

At the OTU level (Figure 3d), the characteristic OTUs were determined through LEfSe analysis in the mucosa-associated microbiota of Hu sheep. Our analysis indicated that 20 OTUs were significantly affected by the treatments (*p* < 0.05). As for the impact of the HG diet, which influenced a total of 13 OTUs, was most apparent among these three groups, as indicated by OTU4 (G: *Vibrionimonas*), OTU5 (G: *Acetitomaculum*), OTU16 (G: *Candidatus Saccharimonas*), OTU205 (G: *Reyranella*), and so on (*p* < 0.05). Five OTUs, including OTU22 (G: *Olsenella*), OTU54 (G: *Pseudomonas*), OTU84 (G: *Stenotrophomonas*), OTU116 (G: *Stenotrophomonas*), and OTU229 (S: *Eimeria praecox*) were enriched (*p* < 0.05) in the CON group. The HP group was enriched (*p* < 0.05) significantly by OTU92 (O: *RF39*) and OTU26 (Unclassified).

### 3.5. Changes in the Microbial Functions of Jejunal Microbiota

**Figure 4:**
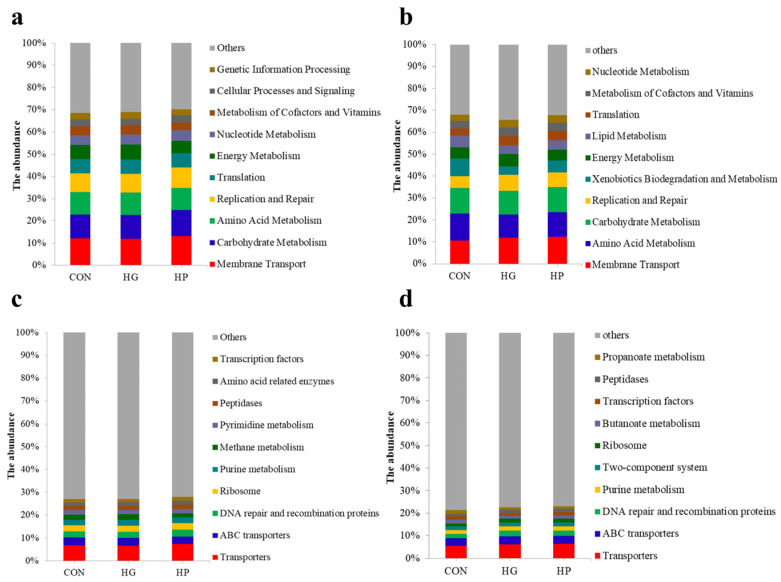
The metabolic profiles of jejunal digesta- and mucosa-associated microbiota at the KEGG level 2 (**a**,**b**) and 3 (**c**,**d**) in Hu sheep (n = 5).

To increase understanding of the microbial changes in the jejunum after the supplementation of different diets, we analyzed the potential functions of jejunal microbiota. At KEGG level 2 (Figure 4a,b), metabolic functions such as membrane transport, carbohydrate metabolism, and amino acid metabolism were dominant in both digesta- and mucosa-associated microbiota. At KEGG level 3 (Figure 4c,d), transporters, ABC transporters, DNA repair, and recombination proteins were dominant in both digesta- and mucosa-associated microbiota.

**Figure 5:**
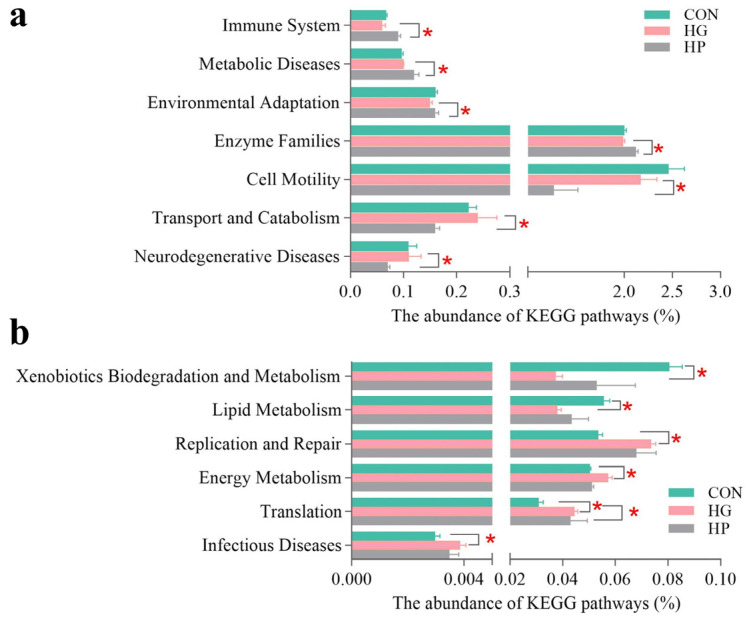
The alterations in the metabolic functions of jejunal digesta- (**a**) and mucosa- (**b**) associated microbiota at the KEGG level 2 in Hu sheep (n = 5) fed the CON, HG, and HP diets. CON, the control group; HG, the high-grain group; HP, the pelleted high-grain group. * *p* < 0.05.

**Figure 6:**
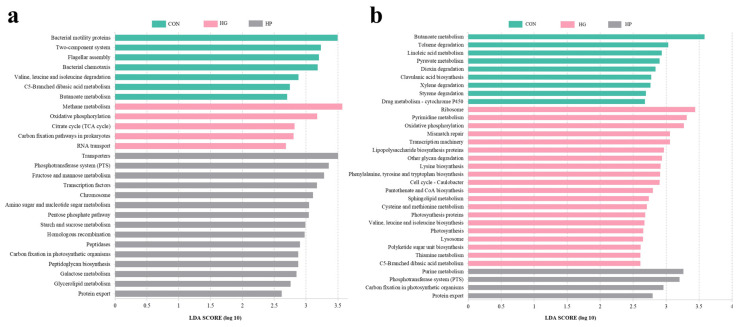
The alterations in the metabolic functions of jejunal digesta- (**a**) and mucosa- (**b**) associated microbiota at the KEGG level 3 in Hu sheep (n = 5) fed the CON, HG, and HP diets. CON, the control group; HG, the high-grain group; HP, the pelleted high-grain group.

Next, we investigated the specific impacts of different diets on the jejunal microbial functions of Hu sheep. For digesta-associated microbiota, at KEGG level 2 (Figure 5a), an HP diet significantly increased the abundance of environmental adaptation (*p* = 0.025), enzyme families (*p* = 0.032), immune system (*p* = 0.021), and metabolic diseases (*p* = 0.021), and significantly decreased the abundance of cell motility (*p* = 0.036), neurodegenerative diseases (*p* = 0.032), and transport and catabolism (*p* = 0.016), as compared with the HG diets. Subsequently, we performed the LEfSe analysis of microbial functions at KEGG level 3 (Figure 6a), the results showed that 27 metabolic pathways differed significantly depending on the diet (*p* < 0.05). Of these, seven pathways (for instance, bacterial motility proteins, bacterial chemotaxis, butanoate metabolism, and so on) were featured (*p* < 0.05) in the CON group. There are five pathways (for example, methane metabolism, oxidative phosphorylation, citrate cycle (TCA cycle), and so on) were featured (*p* < 0.05) in the HG group. The remaining 15 pathways, such as amino sugar and nucleotide sugar metabolism, starch and sucrose metabolism, and galactose metabolism were featured (*p* < 0.05) in the HP group.

For the jejunal mucosa, concerning pathway-specific differences at the KEGG level 2 (Figure 5b), our results found the HG diet decreased the abundance of the xenobiotics biodegradation and metabolism (*p* = 0.004), and the lipid metabolism (*p* = 0.007) pathways, while increased the abundance of infectious diseases (*p* = 0.015), replication and repair (*p* = 0.005), energy metabolism (*p* = 0.013), and the translation (*p* = 0.004) pathways, compared to the CON group. Moreover, there was decreased abundance in terms of the energy metabolism (*p* = 0.002) pathway in the HP group compared to the HG group. At KEGG level 3 (Figure 6b), the results of LEfSe analysis showed that 33 pathways were significantly affected by dietary treatment (*p* < 0.05). Among them, 20 pathways, including ribosome, pyrimidine metabolism, and oxidative phosphorylation were featured (*p* < 0.05) in the HG group; nine pathways, such as butanoate metabolism, toluene degradation, linoleic acid metabolism, and so on, were enriched (*p* < 0.05) in the CON group; four pathways, such as carbon fixation in photosynthetic organisms, phosphotransferase system (PTS), and purine metabolism were gathered (*p* < 0.05) in the HP group.

## 4. Discussion

The effects of high-grain diets on improving ruminant performance has been well documented [1,2]. Indeed, our previous study showed that the HG diets significantly increased the average daily gain and body weight gain of Hu sheep [14]. Although the nutrient intakes of Hu sheep in the HP group was lower than that in the HG group, there were no significant differences in the growth performance between the HG and HP groups [14]. Except for growth performance, whether the HP diets can replace the HG diets in ruminant rearing still requires a series of comprehensive evaluations. In this study, we investigated the effects of HG and HP diets on small intestine from the perspective of intestinal microbiota. Our findings contribute to enriching the information about the high-grain diets and also provide favorable evidence for the application of HP diets in ruminant rearing.

### 4.1. Dietary Treatments Led to Differential Response Changes in Microbial Richness and Diversity in Different Ecological Niches

Maintaining a certain degree of microbial richness and diversity is essential to the stability of the intestinal micro-ecological environment and host health [27,28]. Our study data suggested that both HG and HP diets significantly reduced the microbial richness and diversity in the jejunal digesta, as indicated by the lower Chao 1 and Shannon index value for the digesta-associated microbiota of the HG group (HG vs. CON) and HP group (HP vs. HG). These observations were consistent with previous studies that confirm that an HG diet reduced the richness and diversity of digesta-associated microbiota in the gastrointestinal tracts of ruminants (goats and dairy cows), including the rumen, the cecum, and the colon [29,30,31]. The above changes were related to a large amount of undigested starch entering into the jejunum of the animals in the HG group. However, contrary to the significant differences in digesta-associated microbiota, only a minor difference (the lower Chao 1 index value for the HP group than the HG group) was observed in the mucosa-associated microbiota after the different dietary treatments, indicating more stable microbial diversity in mucosa-associated versus digesta-associated microbiota.

### 4.2. Changes in the Microbial Structure of Jejunal Digesta after Different Dietary Treatments

The PCoA and PERMANOVA of jejunal digesta-associated microbiota showed that a significant difference between the HP and HG groups only. Regarding specific differences in microbial phyla, the HG group considerably decreased the abundance of Firmicutes in the jejunal digesta, which was consistent with the findings of studies investigating the distal jejunum of dairy cows fed a grain-based diet [32]. The phylum Firmicutes also occupied an absolutely dominant position in the jejunal digesta investigated in the present study and in other intestinal regions of ruminants [22,31], highlighting their important role in carbohydrate degradation. Moreover, our results also found that the abundance of Verrucomicrobiota was significantly lower in the HP group than in the HG group. A metagenomic study on the ruminal microbiome revealed that the assembled genomes belonging to Verrucomicrobiota were enriched in terms of genes related to cellulose degradation [33]. The lower nutrient intakes of neutral detergent fiber and acid detergent fiber in the HP diets may be one of the reasons the HP diet reduced the abundance of Verrucomicrobiota in the jejunal digesta in this study.

At a lower taxonomic level, our results showed that some acid-producing bacteria, including the genus *Bifidobacterium*, OTU151 (G: *Acetitomaculum*), and OTU16 (G: *Candidatus Saccharimonas*), were enriched in the jejunal digesta of Hu sheep fed HG diets. *Bifidobacterium* is a widely recognized intestinal probiotic that has some health significance for both animals and humans, and it works by fermenting various sugars to acidic substances, such as lactic acid and propionate [34,35]. Strains of *Acetitomaculum* were obligate anaerobe and fermented various substrates (such as glucose, fructose, and cellobiose) to acetate in the rumen of ruminants [36,37]. The evidence indicated that the ruminal *Candidatus Saccharimonas* was significantly and positively correlated with proportion production [38], and a significant increase in these microorganisms in the rumen of dairy cows suffering from laminitis has been reported [39]. Meanwhile, we also found a significant decrease in some beneficial microorganisms, such as *Christensenellaceae R-7 group* and *Blautia*, in the animals in the HG diets group compared to those in the CON group. Bacteria from *Christensenellaceae R-7 group* were strictly anaerobic and Gram-negative and generated acetate and butyrate as their fermentation end products [40]. A meta-analysis of human intestinal diseases showed that *Christensenellaceae R-7 group* was significantly connected with the state of health of the test subjects [41]. *Blautia* are anaerobic organisms with probiotic roles that often appear in the feces and intestines of mammals, and its reduction in the intestine has a significant correlation with some inflammatory diseases [42].

In addition, we observed an increase in the abundance of acid-producing taxons, including *Olsenella*, *Pseudoramibacter*, and *Shuttleworthia*, while there was a reduction in the abundance of pro-inflammatory taxons, including *Mogibacterium* and *Marvinbryantia* after been fed an HP diet compared to an HG diet. Similar to our results, a higher proportion of *Olsenella* in the ruminal content and jejunal mucosa of ruminants after being fed an HG diet has been reported in previous studies on laying hens [43,44]. *Olsenella* belonging to *Actinobacteria* has the ability to ferment carbohydrates to volatile fatty acids (VFAs), such as acetate and butyrate [45], and is also considered to help boost the anti-inflammatory capacity of a host in a goat model [45]. A previous study on Holstein steers and goats showed that increased grain feeding significantly elevated the abundance of *Shuttleworthia* in the rumen [43] and of *Pseudoramibacter* in the cecum [46], respectively, and these two genera were considered butyrate-producing microorganisms in a study of Holstein heifers and humans [47,48]. Moreover, *Mogibacterium* was reported to be more prevalent in the intestines of colorectal cancer patients than in healthy controls [49], and the genus *Marvinbryantia* was proven to be linked with intestinal inflammation in stress models (mice and humans) [50,51]. Therefore, the above results suggest that the decreased abundance of intestinal *Mogibacterium* and *Marvinbryantia* during HP feeding may partially protect intestinal health.

### 4.3. Changes of the Microbial Community in Jejunal Mucosa-Associated Microbiota after Various Dietary Treatments

As the digesta- and mucosa-associated microbiota differs in their structure and abundance and the mucosa-associated microbiota is also in contact with the host directly [52,53], it is therefore also important to investigate the microbial structure of mucosal niches. PCoA and PERMANOVA analyses revealed significant differences in the jejunal mucosa-associated microbiota of the animals in the CON and HG groups, and also the HG and HP groups in the present study. For specific differences, among the dominant phyla, the HG diets reduced the abundance of *Actinobacteriota* in the jejunal mucosa compared to the CON diet, and both the HG and HP diets reduced the abundance of *Bacteroidetes* in the jejunal mucosa compared to the CON or HG diets. These results indicate that different treatments had a huge impact on the mucosa-associated microbiota of Hu sheep in the present study.

Regarding the genus-specific distinction in the jejunum mucosal microbiota, our results suggested that the abundance of some acid-producing bacteria (*Rikenellaceae RC9 gut group* and *Bifidobacterium*) and pro-inflammatory bacteria (*Erysipelotrichaceae UCG-009* and *Mycobacterium*) increased in the HG group compared to the CON group. The *Rikenellaceae RC9 gut group*, members of the family *Rikenellaceae*, was considered to be associated with carbohydrate degradation, and some *Rikenellaceae* spp. were producers of VFAs, such as acetate and propionate [54,55]. However, contrary to the results of one previous study on the rumen [54], we observed a significant increase in its abundance in the HG group (compared to the CON group) and a significant decrease in the HP group (compared to the HG group). Because the family *Rikenellaceae* is a relatively new taxonomic group, the exact role of the *Rikenellaceae RC9 gut group* in adapting to HG diets still requires further study. Not surprisingly, due to their acid-producing capacity, consistent with the changes in the content, a prominent increase in the abundance of *Bifidobacterium* was found in the jejunal mucosa in this study. Moreover, there was substantial evidence supporting the association between inflammation-related gastrointestinal diseases and bacterial strains in these two genera (*Erysipelotrichaceae* and *Mycobacterium*) [49,56,57]. Therefore, the increased abundance of *Erysipelotrichaceae UCG-009* and *Mycobacterium* in the jejunal mucosa in the HG group may have some adverse effects on intestinal fitness, such as local inflammation.

Our results also showed a significantly lower abundance of *Prevotella*, *Chitinophaga*, and *Candidatus Saccharimonas* in the jejunal mucosa of the HP group compared to the HG group. *Prevotella* members are reported to have a variety of metabolic functions and to participate in the degradation of multiple substances, such as starch, fiber, and protein [58,59]. Therefore, this change in the abundance of *Prevotella* may be a substrate-dependent phenomenon that occurs in the mucosa of sheep after being after an HP diet. Moreover, the pathogenic potential of *Chitinophaga* spp. was confirmed in a previous study, and always isolated from some human clinical subjects [60]. In addition, *Candidatus Saccharimonas* were found to proliferate in the intestinal lumen of parasite-infected sheep [61] and rumen of dairy cows suffering from laminitis [39]. Therefore, consistent with the changes observed in the digesta-associated microbiota, these results showed that an HP diet seemed to be able to partially alleviate the adverse effects of HG diets.

### 4.4. Alterations in Jejunal Microbiota Resulted in Distinct Microbial Functions after Different Diets

Our results on microbial functions (at KEGG level 2) showed that, carbohydrate metabolism and amino acid metabolism were predominant in both jejunal digesta- and mucosa-associated microbiota. This result reconfirmed the previous point that the metabolic functions of intestinal microbiota essential for survival are carbohydrate and amino acid metabolism. This result was the same as the results in related research on dairy cows [22] and goats [62]. Regarding the functional differences in jejunal digesta-associated microbiota caused by the dietary treatments, our results indicated that three pathways related to energy metabolism (included citrate cycle (TCA cycle), oxidative phosphorylation, and methane metabolism) were enriched in the HG group. The TCA cycle is the essential metabolic pathway of three major nutrients (carbohydrate, lipids, and amino acids) and releases energy for the host [63]; oxidative phosphorylation is the primary source of ATP generation that supports cell growth, and this process requires the participation of NADH produced by TCA [64]; and methane formation is an important route of energy loss in ruminants. These increases corresponded to the rich nutrients and higher energy levels in the HG diet. Moreover, our results also showed that the pathways related to simple sugars (including galactose metabolism; starch and sucrose metabolism; amino sugar and nucleotide sugar; and fructose and mannose metabolism) were enriched in the HP group. Previous related research revealed that an HP diet promoted the simple sugars utilized by ruminal microorganisms [14,21], and our above-mentioned results are consistent with this. This may be related to the increased simple sugars in HP diets through pelleting.

Interestingly, our results showed that the pathway lipopolysaccharide (LPS) biosynthesis proteins was featured in the jejunal mucosa of the HG group. This indicates that the ability of jejunal microbiota to synthesize LPS may be enhanced after being fed an HG diet feeding. It is well documented that an HG diet increases the concentration of LPS in the gastrointestinal tract of ruminants, such as in the rumen, the cecum, and the colon [6,65], and the release of LPS is associated with dramatic changes in the amount of Gram-negative microbiota [66]. Corresponding to this, our data indicated that several pathways related to amino acids biosynthesis (including lysine biosynthesis; phenylalanine; tyrosine and tryptophan biosynthesis; cysteine and methionine metabolism; valine; and leucine and isoleucine biosynthesis) were enriched in the HG group. These results partly suggest that an HG diet leads to a higher turnover rate of jejunal mucosa-associated microbiota in Hu sheep. Moreover, the jejunal mucosa-associated microbiota was only enriched by four pathways in the HP group. Among them, the PTS was used by intestinal microbiota for carbohydrate uptake, particularly hexoses, hexitols, and disaccharides [67]. Thus, compared with the digesta-associated microbiota, although the functional changes in the mucosa-associated microbiota in the HP group were smaller, the mucosa-associated microbiota also enhanced the utilization of simple sugars.

## 5. Conclusions

In conclusion, our study revealed the differential responses of digesta- and mucosa-associated jejunal microbiota to HG and HP diets in Hu sheep. Our results demonstrated that the HG diets promoted the proliferation of acid-producing bacteria in both jejunal digesta and mucosa compared with the CON diets. In addition to the similar effects of the HG diets, the HP diets reduced the proliferation of some pro-inflammatory bacteria in the jejunal digesta and mucosa of Hu sheep compared to the HG diets. Overall, our results based on intestinal microbiota showed that the application effect of HP diets was better than HG diets. These findings contributed to enriching the knowledge about the impact of high-grain diets on the structure and function of intestinal microbiota in ruminants, and also provide favorable evidence for the application of HP diets in ruminant rearing. Nevertheless, more studies should center around the impacts of HP and HG diets on host health, including on intestinal morphology, gene expression, and mucosal immunity.

## Data Availability

In the present study, the 16S rRNA gene sequencing data of digesta- and mucosa-associated microbiota in the jejunum had been submitted to National Center for Biotechnology Information Sequence Read Archive under the accession number PRJNA781219 (accessed on 18 November 2021).

## Supplementary Materials

The following supporting information can be downloaded at: https://www.mdpi.com/article/10.3390/ani12131695/s1, Figure S1: The rarefaction curves generated from the digest- (**a**) and mucosa-associated (**b**) microbiota in the jejunum of Hu sheep; Table S1: Ingredients, proximate analysis, and nutrients intake of non-pelleted low-grain diets (CON), non-pelleted high-grain diets (HG) and pelleted (HP) high-grain diets.

## References

[1] Krause K., Oetzel G. (2005). Inducing subacute ruminal acidosis in lactating dairy cows. J. Dairy Sci..

[2] Gao X., Oba M. (2014). Relationship of severity of subacute ruminal acidosis to rumen fermentation, chewing activities, sorting behavior, and milk production in lactating dairy cows fed a high-grain diet. J. Dairy Sci..

[3] Liu J.-H., Bian G.-R., Zhu W.-Y., Mao S.-Y. (2015). High-grain feeding causes strong shifts in ruminal epithelial bacterial community and expression of Toll-like receptor genes in goats. Front. Microbiol..

[4] Hua C., Tian J., Tian P., Cong R., Luo Y., Geng Y., Tao S., Ni Y., Zhao R. (2017). Feeding a high concentration diet induces unhealthy alterations in the composition and metabolism of ruminal microbiota and host response in a goat model. Front. Microbiol..

[5] Zhang R., Jin W., Feng P., Liu J., Mao S. (2018). High-grain diet feeding altered the composition and functions of the rumen bacterial community and caused the damage to the laminar tissues of goats. Animal.

[6] Metzler-Zebeli B.U., Schmitz-Esser S., Klevenhusen F., Podstatzky-Lichtenstein L., Wagner M., Zebeli Q. (2013). Grain-rich diets differently alter ruminal and colonic abundance of microbial populations and lipopolysaccharide in goats. Anaerobe.

[7] Wang Y., Xu L., Liu J., Zhu W., Mao S. (2017). A high grain diet dynamically shifted the composition of mucosa-associated microbiota and induced mucosal injuries in the colon of sheep. Front. Microbiol..

[8] Ye H., Liu J., Feng P., Zhu W., Mao S. (2016). Grain-rich diets altered the colonic fermentation and mucosa-associated bacterial communities and induced mucosal injuries in goats. Sci. Rep..

[9] Bonfante E., Palmonari A., Mammi L., Canestrari G., Fustini M., Formigoni A. (2016). Effects of a completely pelleted diet on growth performance in Holstein heifers. J. Dairy Sci..

[10] Owens F.N., Secrist D.S., Hill W.J., Gill D.R. (1997). The effect of grain source and grain processing on performance of feedlot cattle: A review. J. Anim. Sci..

[11] Blanco C., Giráldez F.J., Prieto N., Benavides J., Wattegedera S., Morán L., Andrés S., Bodas R. (2015). Total mixed ration pellets for light fattening lambs: Effects on animal health. Animal.

[12] Zhong R., Fang Y., Zhou D., Sun X., Zhou C., He Y. (2018). Pelleted total mixed ration improves growth performance of fattening lambs. Anim. Feed Sci. Technol..

[13] Li B., Sun X., Huo Q., Zhang G., Wu T., You P., He Y., Tian W., Li R., Li C. (2021). Pelleting of a total mixed ration affects growth performance of fattening lambs. Front. Vet. Sci..

[14] Trabi E.B., Seddik H.-e., Xie F., Lin L., Mao S. (2019). Comparison of the rumen bacterial community, rumen fermentation and growth performance of fattening lambs fed low-grain, pelleted or non-pelleted high grain total mixed ration. Anim. Feed Sci. Technol..

[15] Lin L., Trabi E.B., Xie F., Mao S. (2021). Comparison of the fermentation and bacterial community in the colon of Hu sheep fed a low-grain, non-pelleted, or pelleted high-grain diet. Appl. Microbiol. Biotechnol..

[16] Myer P., Wells J., Smith T., Kuehn L., Freetly H. (2016). Microbial community profiles of the jejunum from steers differing in feed efficiency. J. Anim. Sci..

[17] Zhang X., Wu J., Zhou C., Tan Z., Jiao J. (2021). Spatial and temporal organization of jejunal microbiota in goats during animal development process. J. Appl. Microbiol..

[18] Wen K., Zhao M., Liu L., Khogali M.K., Geng T., Wang H., Gong D. (2021). Thiamine modulates intestinal morphological structure and microbiota under subacute ruminal acidosis induced by a high-concentrate diet in Saanen goats. Animal.

[19] Yuan C., Graham M., Staley C., Subramanian S. (2020). Mucosal microbiota and metabolome along the intestinal tract reveal a location-specific relationship. Msystems.

[20] Malmuthuge N., Griebel P.J., Guan L.L. (2014). Taxonomic identification of commensal bacteria associated with the mucosa and digesta throughout the gastrointestinal tracts of preweaned calves. Appl. Environ. Microbiol..

[21] Trabi E.B., Seddik H., Xie F., Wang X., Liu J., Mao S. (2020). Effect of pelleted high-grain total mixed ration on rumen morphology, epithelium-associated microbiota and gene expression of proinflammatory cytokines and tight junction proteins in Hu sheep. Anim. Feed Sci. Technol..

[22] Mao S., Zhang M., Liu J., Zhu W. (2015). Characterising the bacterial microbiota across the gastrointestinal tracts of dairy cattle: Membership and potential function. Sci. Rep..

[23] Magoč T., Salzberg S.L. (2011). FLASH: Fast length adjustment of short reads to improve genome assemblies. Bioinformatics.

[24] Edgar R.C., Haas B.J., Clemente J.C., Quince C., Knight R. (2011). UCHIME improves sensitivity and speed of chimera detection. Bioinformatics.

[25] Langille M.G., Zaneveld J., Caporaso J.G., McDonald D., Knights D., Reyes J.A., Clemente J.C., Burkepile D.E., Thurber R.L.V., Knight R. (2013). Predictive functional profiling of microbial communities using 16S rRNA marker gene sequences. Nat. Biotechnol..

[26] Caporaso J.G., Kuczynski J., Stombaugh J., Bittinger K., Bushman F.D., Costello E.K., Fierer N., Peña A.G., Goodrich J.K., Gordon J.I. (2010). QIIME allows analysis of high-throughput community sequencing data. Nat. Methods.

[27] Consortium H.M.P. (2012). Structure, function and diversity of the healthy human microbiome. Nature.

[28] Tuddenham S., Sears C.L. (2015). The intestinal microbiome and health. Curr. Opin. Infect. Dis..

[29] Fernando S.C., Purvis H., Najar F., Sukharnikov L., Krehbiel C., Nagaraja T., Roe B., Desilva U. (2010). Rumen microbial population dynamics during adaptation to a high-grain diet. Appl. Environ. Microbiol..

[30] Plaizier J.C., Li S., Tun H.M., Khafipour E. (2017). Nutritional models of experimentally-induced subacute ruminal acidosis (SARA) differ in their impact on rumen and hindgut bacterial communities in dairy cows. Front. Microbiol..

[31] Liu J., Xu T., Zhu W., Mao S. (2014). High-grain feeding alters caecal bacterial microbiota composition and fermentation and results in caecal mucosal injury in goats. Br. J. Nutr..

[32] Plaizier J.C., Danscher A.-M., Azevedo P.A., Derakhshani H., Andersen P.H., Khafipour E. (2021). A Grain-Based SARA Challenge Affects the Composition of Epimural and Mucosa-Associated Bacterial Communities throughout the Digestive Tract of Dairy Cows. Animals.

[33] Gharechahi J., Vahidi M.F., Bahram M., Han J.-L., Ding X.-Z., Salekdeh G.H. (2021). Metagenomic analysis reveals a dynamic microbiome with diversified adaptive functions to utilize high lignocellulosic forages in the cattle rumen. ISME J..

[34] Bunesova V., Vlkova E., Rada V., Killer J., Musilova S. (2014). Bifidobacteria from the gastrointestinal tract of animals: Differences and similarities. Benef. Microbes.

[35] Pokusaeva K., Fitzgerald G.F., van Sinderen D. (2011). Carbohydrate metabolism in Bifidobacteria. Genes Nutr..

[36] Rainey F.A. (2015). Acetitomaculum. Bergey’s Manual of Systematics of Archaea and Bacteria.

[37] Greening R., Leedle J. (1989). Enrichment and isolation of Acetitomaculum ruminis, gen. nov., sp. nov.: Acetogenic bacteria from the bovine rumen. Arch. Microbiol..

[38] Tong J., Zhang H., Yang D., Zhang Y., Xiong B., Jiang L. (2018). Illumina sequencing analysis of the ruminal microbiota in high-yield and low-yield lactating dairy cows. PLoS ONE.

[39] Guo J., Mu R., Li S., Zhang N., Fu Y., Hu X. (2021). Characterization of the Bacterial Community of Rumen in Dairy Cows with Laminitis. Genes.

[40] Fan Q., Wanapat M., Hou F. (2021). Rumen bacteria influence milk protein yield of yak grazing on the Qinghai-Tibet plateau. Anim. Biosci..

[41] Mancabelli L., Milani C., Lugli G.A., Turroni F., Cocconi D., van Sinderen D., Ventura M. (2017). Identification of universal gut microbial biomarkers of common human intestinal diseases by meta-analysis. FEMS Microbiol. Ecol..

[42] Liu X., Mao B., Gu J., Wu J., Cui S., Wang G., Zhao J., Zhang H., Chen W. (2021). Blautia—A new functional genus with potential probiotic properties?. Gut Microbes.

[43] Plaizier J., Azevedo P., Schurmann B., Górka P., Penner G., Khafipour E. (2020). The duration of increased grain feeding affects the microbiota throughout the digestive tract of yearling Holstein steers. Microorganisms.

[44] Gaowa N., Li W., Gelsinger S., Murphy B., Li S. (2021). Analysis of Host Jejunum Transcriptome and Associated Microbial Community Structure Variation in Young Calves with Feed-Induced Acidosis. Metabolites.

[45] Wang W.-W., Jia H.-J., Zhang H.-J., Wang J., Lv H.-Y., Wu S.-G., Qi G.-H. (2019). Supplemental plant extracts from Flos lonicerae in combination with Baikal skullcap attenuate intestinal disruption and modulate gut microbiota in laying hens challenged by Salmonella pullorum. Front. Microbiol..

[46] Tao S., Tian P., Luo Y., Tian J., Hua C., Geng Y., Cong R., Ni Y., Zhao R. (2017). Microbiome-metabolome responses to a high-grain diet associated with the hind-gut health of goats. Front. Microbiol..

[47] O’Hara E., Kelly A., McCabe M.S., Kenny D.A., Guan L.L., Waters S.M. (2018). Effect of a butyrate-fortified milk replacer on gastrointestinal microbiota and products of fermentation in artificially reared dairy calves at weaning. Sci. Rep..

[48] Kong F., Gao Y., Tang M., Fu T., Diao Q., Bi Y., Tu Y. (2020). Effects of dietary rumen–protected Lys levels on rumen fermentation and bacterial community composition in Holstein heifers. Appl. Microbiol. Biotechnol..

[49] Chen W., Liu F., Ling Z., Tong X., Xiang C. (2012). Human intestinal lumen and mucosa-associated microbiota in patients with colorectal cancer. PLoS ONE.

[50] Wang Y., Xie Q., Sun S., Huang B., Zhang Y., Xu Y., Zhang S., Xiang H. (2018). Probiotics-fermented Massa Medicata Fermentata ameliorates weaning stress in piglets related to improving intestinal homeostasis. Appl. Microbiol. Biotechnol..

[51] Zhang Y., Chen L., Hu M., Kim J.J., Lin R., Xu J., Fan L., Qi Y., Wang L., Liu W. (2020). Dietary type 2 resistant starch improves systemic inflammation and intestinal permeability by modulating microbiota and metabolites in aged mice on high-fat diet. Aging.

[52] Carroll I.M., Chang Y.-H., Park J., Sartor R.B., Ringel Y. (2010). Luminal and mucosal-associated intestinal microbiota in patients with diarrhea-predominant irritable bowel syndrome. Gut Pathog..

[53] Heinsen F.-A., Knecht H., Neulinger S.C., Schmitz R.A., Knecht C., Kühbacher T., Rosenstiel P.C., Schreiber S., Friedrichs A.K., Ott S.J. (2015). Dynamic changes of the luminal and mucosa-associated gut microbiota during and after antibiotic therapy with paromomycin. Gut Microbes.

[54] Qiu Q., Gao C., Aziz ur Rahman M., Cao B., Su H. (2020). Digestive ability, physiological characteristics, and rumen bacterial community of holstein finishing steers in response to three nutrient density diets as fattening phases advanced. Microorganisms.

[55] Holman D.B., Yang W., Alexander T.W. (2019). Antibiotic treatment in feedlot cattle: A longitudinal study of the effect of oxytetracycline and tulathromycin on the fecal and nasopharyngeal microbiota. Microbiome.

[56] Kaakoush N.O. (2015). Insights into the role of Erysipelotrichaceae in the human host. Front. Cell. Infect. Microbiol..

[57] Davis W.C., Madsen-Bouterse S.A. (2012). Crohn’s disease and Mycobacterium avium subsp. paratuberculosis: The need for a study is long overdue. Vet. Immunol. Immunopathol..

[58] Ntaikou I., Gavala H.N., Kornaros M., Lyberatos G. (2008). Hydrogen production from sugars and sweet sorghum biomass using Ruminococcus albus. Int. J. Hydrogen Energy.

[59] Petri R.M., Schwaiger T., Penner G.B., Beauchemin K.A., Forster R.J., McKinnon J.J., McAllister T.A. (2013). Characterization of the core rumen microbiome in cattle during transition from forage to concentrate as well as during and after an acidotic challenge. PLoS ONE.

[60] Tran T.L.Q., Anani H., Trinh H.T., Pham T.P.T., Ho V.M., Bui N.H.L., Nguyen N.H., Raoult D., Trinh T.T., Fournier P.E. (2020). *Chitinophaga vietnamensis* sp. nov., a multi-drug resistant bacterium infecting humans. Int. J. Syst. Evol. Microbiol..

[61] Cortés A., Wills J., Su X., Hewitt R.E., Robertson J., Scotti R., Price D.R., Bartley Y., McNeilly T.N., Krause L. (2020). Infection with the sheep gastrointestinal nematode Teladorsagia circumcincta increases luminal pathobionts. Microbiome.

[62] Jiao J., Zhang X., Wang M., Zhou C., Yan Q., Tan Z. (2018). Linkages between epithelial microbiota and host transcriptome in the ileum during high-grain challenges: Implications for gut homeostasis in goats. J. Agric. Food Chem..

[63] Dashty M. (2013). A quick look at biochemistry: Carbohydrate metabolism. Clin. Biochem..

[64] Wilson D.F. (2017). Oxidative phosphorylation: Regulation and role in cellular and tissue metabolism. J. Physiol..

[65] Li S., Khafipour E., Krause D., Kroeker A., Rodriguez-Lecompte J., Gozho G., Plaizier J. (2012). Effects of subacute ruminal acidosis challenges on fermentation and endotoxins in the rumen and hindgut of dairy cows. J. Dairy Sci..

[66] Nagaraja T., Bartley E., Fina L., Anthony H. (1978). Relationship of rumen gram-negative bacteria and free endotoxin to lactic acidosis in cattle. J. Anim. Sci..

[67] Siebold C., Flükiger K., Beutler R., Erni B. (2001). Carbohydrate transporters of the bacterial phosphoenolpyruvate: Sugar phosphotransferase system (PTS). FEBS Lett..

