# Supplementary material for: Differential Responses of Digesta- and Mucosa-Associated Jejunal Microbiota of Hu Sheep to Pelleted and Non-Pelleted High-Grain Diets
Source: Animals (Basel). 2022 Jun 30;12(13):1695. doi: 10.3390/ani12131695 (PMC9264909; doi:10.3390/ani12131695)
Supplement: Supplementary file 1 [file animals-12-01695-s001.zip › animals-1692801-supplementary.pdf]

animals

MDPI

Supplementary Materials

Differential Responses of Digesta- and Mucosa-Associated Jejunal Microbiota of Hu Sheep to Pelleted and Non-Pelleted High-Grain Diets

Zhiqiang Zhong †, Yuning Zhang †, Xiaotong Li, Lingyun Li, Ruiyang Zhang * and Shuyi Zhang *

College of Animal Science and Veterinary Medicine, Shenyang Agricultural University, Shenyang 110866, China; zhongzhiqiang@stu.syau.edu.cn (Z.Z.); zhangyuning@stu.syau.edu.cn (Y.Z.); lixiaotong20221019@163.com (X.L.); lilingyun@stu.syau.edu.cn (L.L.)
*   Correspondence: zhangruiyang@syau.edu.cn (R.Z.); szhang@syau.edu.cn (S.Z.)
†   These authors contributed equally to this work.

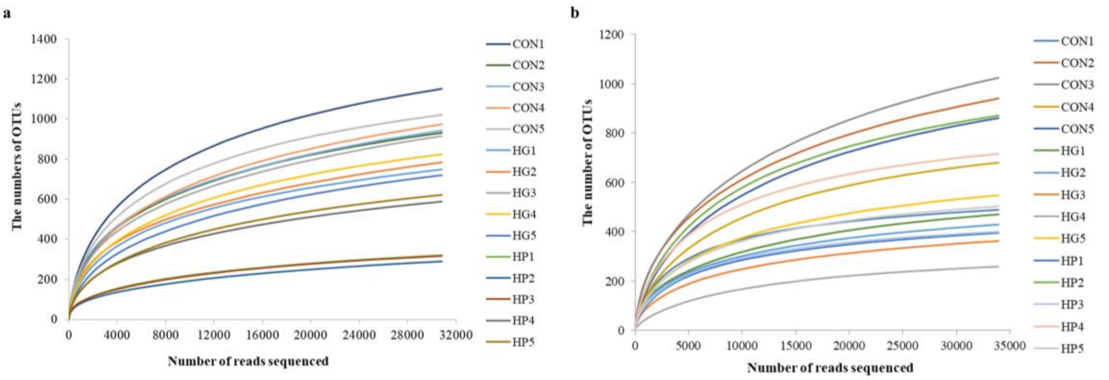

Figure S1. The rarefaction curves generated from the digesta- (**a**) and mucosa-associated (**b**) microbiota in the jejunum of Hu sheep.

Table S1. Ingredients, proximate analysis, and nutrients intake of non-pelleted low-grain diets (CON), non-pelleted high-grain diets (HG) and pelleted (HP) high-grain diets.

Item	CON	HG	HP
**Ingredients,(g/kg)DM**			
Oat straw	520	230	230
Alfalfa hay	180	70.0	70.0
Corn	192	406	406
Wheat bran	-	156	156
Soybean meal	67.0	90.0	90.0
Stone powder	5.00	17.5	17.5
Calcium hydrogen phosphate	9.00	3.50	3.50
Zeolite powder	10.0	10.0	10.0
Salt	7.00	7.00	7.00
Trace mineral salt and vitamins [a]	10.0	10.0	10.0
**Nutrient composition**			
Metabolic energy (MJ/kg) [b]	8.92	9.89	9.89
Crude protein (g/kg)	139	138	138
Neutral detergent fibre (g/kg)	406	267	265
Acid detergent fibre (g/kg)	260	147	146
Indigestible neutral detergent fibre (g/kg)	182	123	123
Indigestible acid detergent fibre (g/kg)	114	77.6	75.6

Nitrogen fractions associated to the fibre (g/kg)	52.0	44.0	42.9
Ether extract (g/kg)	36.0	34.6	33.4
Ash (g/kg)	81.8	64.5	65.2
**Nutrients intake**			
Metabolic energy (MJ/animal/day)	133	152	139
Crude protein (g/animal/day)	207	213	194
Neutral detergent fibre (g/animal/day)	607	413	373
Acid detergent fibre (g/animal/day)	390	227	205
Ether extract (g/animal/day)	53.5	53.1	46.6
Ash (g/animal/day)	122	98.9	90.9

[a] Trace mineral salt and vitamins were purchased from Continental Grain Crop. (Nanjing, China) and contained (per kg of premix) 22,000 IU vitamin A, 5000 IU vitamin E, 100 mg Fe, and 20 mg Cu.
[b] Calculated value.
